# Gas-phase fractionation DDA promotes in-depth DIA phosphoproteome analysis

**DOI:** 10.1016/j.heliyon.2025.e41928

**Published:** 2025-01-14

**Authors:** Zhiwei Tu, Yabin Li, Shuhui Ji, Shanshan Wang, Rui Zhou, Gertjan Kramer, Yu Cui, Fei Xie

**Affiliations:** a State Key Laboratory of Medical Proteomics, Beijing Proteome Research Center, National Center for Protein Sciences (Beijing), Beijing Institute of Lifeomics, 102206, Beijing, China; b College of Pulmonary and Critical Care Medicine, Chinese PLA General Hospital, 100048, Beijing, China; c The First Affiliated Hospital of Henan University of Chinese Medicine, 450000, Zhengzhou, Henan, China; d Laboratory for Mass Spectrometry of Biomolecules, Swammerdam Institute for Life Sciences, University of Amsterdam, Science Park 904, 1098 XH, Amsterdam, the Netherlands

**Keywords:** DIA, Phosphoproteome, Gas phase fractionation, GPF DDA, ARDS/ALI

## Abstract

Data-independent acquisition (DIA) is a promising method for quantitative proteomics. Library-based DIA database searching against project-specific data-dependent acquisition (DDA) spectral libraries is the gold standard. These libraries are constructed using material-consuming pre-fractionation two dimensional DDA analysis. The alternative to this is library-free DIA analysis. Limited sample amounts restrict the use of fractionation to build spectral libraries for post-translational modifications (PTMs) DIA analysis. We present the use of gas-phase fractionation (GPF) DDA data to improve the depth of library-free DIA identification for the phosphoproteome, called GPF-DDA hybrid DIA. This method fully utilizes the remnants of samples post-DIA analysis and leverages both library-based and -free DIA database searching. GPF-DDA hybrid DIA analyzes phosphopeptides surplus sample after DIA analysis using a number of DDA injections with each scanning different mass-to-charge (*m*/*z*) windows, instead of preforming traditional off-line fractionation-based DDA. The GPF-DDA data is integrated into the library-free DIA database search to create a hybrid library, enhancing phosphopeptide identification. Two GPF-DDA injections proved to increase 18 % phosphopeptide and 13 % phosphosite identification in HEK293 cell lines, while five injections resulted in up to 28 % phosphopeptide and 21 % phosphosite increases compared to library-free DIA analysis alone. We used GPF-DDA hybrid DIA phosphoproteomics to characterize lung tissue upon direct (smoke induced) and indirect (sepsis induced) acute lung injury (ALI) in mice. The differentially expressed phosphosites (DEPsites) in direct ALI were found in proteins related to mRNA processing and RNA. DEPsites in indirect ALI were enriched in proteins related to microtubule polymerization, positive regulation of microtubule polymerization and fibroblast migration. This study demonstrates that GPF-DDA hybrid DIA analysis workflow can indeed promote depth of DIA analysis of phosphoproteome and could be extended to DIA analysis of other PTMs.

## 1 Introduction

Data-dependent acquisition (DDA) or shot-gun proteomics is one of revolutionary tools in the study of systems biology [1]. Unlike the Top-N strategy applied in DDA proteomics, data-independent acquisition (DIA) attempts to target all available precursors within the isolation window of interest [2,3], and can reach an unprecedented depth of proteome converge. In DDA, each DDA MS/MS contains only one precursor, whereas DIA fragments all precursors within a wide precursor *m*/*z* range which makes the interpretation of DIA MS/MS spectrum much more complicated and commonly, as the gold standard, requires generating comprehensive DDA spectrum libraries for the spectrum interpretation [4]. A high quality spectrum library can be acquired with significant expenditure of sample, time and effort of offline fractionation [5]. However, only a small fraction of a tryptic peptide sample can be enriched as post-translational modified peptides, such as phosphopeptides, and thus hinders the building of offline fractionated DDA library for a large number of cases. However, advancements in development of computational algorithms allows identification of phosphopeptides directly from raw DIA data without need of DDA libraries (library-free DIA or direct DIA) [6,7].

Leftover phosphopeptide sample will usually exist after direct DIA analysis. To maximize the utilization of these leftovers, we introduce a gas-phase fractionation (GPF) DDA hybrid DIA analysis approach to promote the depth of direct DIA analysis of phosphoproteome. The GPF was used to improve peptide detection in samples by injecting the same sample multiple times with each injection scanning different isolation ranges of precursors [[8], [9], [10], [11], [12]]. The leftover phospopeptides were pooled together to make a mixture, which was then analyzed using DDA coupled with GPF. By combining the GPF-DDA library with the predicted library from direct DIA, a hybrid library was created and utilized to reanalyze DIA data. We benchmarked the GPF-DDA hybrid DIA analysis of phosphoproteome using the HEK293 cell line and demonstrated its applicability by characterizing phosphoproteome of lungs upon acute lung injury (ALI) induced by pulmonary direct (e.g., smoke inhalation, pneumonia) and extrapulmonary indirect (e.g., sepsis, pancreatitis) causes in mice.

ARDS/ALI is a clinical syndrome of acute hypoxemic respiratory failure, generally caused by direct or indirect lung injury [13], accounting for 10.4 % of ICU admissions and resulting in a mortality rate of approximately 34.9–46.1 % [14,15]. Despite many years of research, the pathogenesis of ARDS/ALI has not yet been fully elucidated [16]. A comprehensive phosphoproteome comparison of ARDS of two etiologies may provide deeper insight into disease pathophysiology and pave the way for more specific clinical interventions. Lung tissue from ALI mouse models induced by smoke inhalation and sepsis, as well as healthy control were subjected to GPF-DDA hybrid DIA phophoproteome analysis. Nine samples (three replicates for each condition) of DIA and three additional injections of GPF-DDA were conducted. Compared to direct DIA, GPF-DDA hybrid DIA identified 28100 phosphopeptides (123 % of direct DIA), resulting in an identification of 19164 (106 % of direct DIA) phosphosites. 332 phosphosites (DEPsites) were differentially expressed and clustered into six clusters exhibiting similar expression profiles. two clusters were up or down regulated only in smoke-induced ALI, another two clusters were up or down regulated in sepsis-induced ALI and the last two clusters regulated in both smoke- and sepsis-induced ALI. Gene Ontology analysis revealed that proteins of DEPsites in smoke-induced ALI were enriched in mRNA processing and RNA splicing, while proteins of DEPsites in sepsis-induced ALI were enriched in microtubule polymerization, cell migration and fibroblast migration. Proteins of DEPsites in both smoke- and sepsis-induced ALI were enriched in double-strand bread repair via homologous recombination, regulation of protein complex assembly and regulation of I-kappaB kinase/NF-kappaB signaling.

The full usage of all available sample is one of principles to reach the deepest identification of phosphoproteome. Using the GPF-DDA hybrid DIA workflow we can utilize trace residual phosphopeptides to increase the depth of direct DIA analysis of phosphoproteome. This approach could also be expanded to DIA analysis of other PTMs in cases where only trace peptides remain.

## 2 Materials and methods

### 2.1 Cell lines and acute lung injury mouse models

Approximately 10^8^ cells from cell line HEK293 were fresh prepared for phosphpeptide enrichment and stored at −80 °C. Smoke- and sepsis-induced ALI mouse models were established according to Refs. [17,18]. All experimental procedures and animal welfare protocols adhered to the National Institute of Health guidelines for laboratory animal care. Nine male ICR mice (25 ± 2 g, SPF Biotechnology Co. Ltd. Beijing, China) were randomly assigned to three groups (n = 3 for each group). One group served as the healthy control. The second group was exposed in a smoke-producing experimental chamber(1m × 0.8m × 0.6m) with a 3 g smoke bomb ignition. The mice were removed from the cage after 3 min. The lungs were excised after 48 h and stored at −80 °C. Sepsis was induced in the third group through polymicrobial peritonitis using cecal ligation and puncture. The mice were anesthetized via peritoneal injection of 5 % chloral hydrate at a dosage of 60uL/10g body weight. Abdominal hair was disinfected and removed, followed by an incision in the abdomen. The cecum was ligated at the 1/3 position with surgical silk and punctured with an injection needle. The peritoneum and skin were sutured intermittently. additionally, a subcutaneous injection of 50 ml/kg body weight of normal saline was administered to prevent shock. Lungs were excised after 12 h and stored at −80 °C.

### 2.2 Protein digestion and phosphpeptide enrichment

Cells or shredded lung tissue were lysed in a buffer containing 1 % sodium deoxycholate, 100 mM Tris-HCl (PH 8.8), 10 mM tris(2-carboxyethyl)phosphine (TCEP) and 40 mM 2-chloroacetamide (CAA). Ultrasonication was applied to further lyse cells and tissues. The lysate was heat treated at 95 °C for 5 min and then cooled to room temperature by placing on ice. Protein digestion with trypsin was conducted according to the published FASP method [19]. Simply, cell lysates containing approximately 13.9 mg of HEK293 protein or 500 μg lung protein were subjected to FASP digestion using Vivacon® 500 centrifugal units (10,000 MWCO, Item No.: VN01H02, Sartorius, UK). Fourteen centrifugal units were used for HEK293 with each loading up to 1 mg protein. All samples were centrifuged at 14,000 g for 20 min. Following centrifugation steps were performed applying the same conditions. Each protein sample was washed three times with 200 μl of 50 mM ammonium bicarbonate. Then, an additional 150 μl of 50 mM ammonium bicarbonate was added to redissolve the proteins. Trypsin (Promega, Madison, USA) was added at a protease: protein (w/w) ratio of 1:50 for overnight digestion at 37 °C. Peptides were collected by centrifugation, and an additional elution was performed with 100 μl of H2O. Phosphopeptides were enriched using titanium dioxide beads (5 μm Titansphere, GL Sciences, Japan). Vacuum dried peptides were incubated with TiO_2_ beads at a peptide to beads ratio of 1:60 in 600 μl binding buffer (70 % ACN, 5 % TFA, 8.3 % lactate in H_2_O) for 40 min. HEK293 peptides were divided into 10 reactions for enrichment, and the resulting phosphopeptides were pooled for further use. Un-phosphorylated peptides were removed by washing the beads three times with 600 μl of each of two wash buffers (wash buffer 1: 30 % ACN, 5 % TFA; wash buffer 2: 80 % ACN, 5 % TFA). Two elutions were performed using 200 μl of elution buffer (40 % ACN and 18 % ammonium hydroxide). The phosphopeptides were pooled and vacuum dried for LC-MS/MS analysis.

### 2.3 DIA and GPF-DDA LC-MS/MS

An EASY-nLC 1200 UPLC system combined with a Q Exactive HF mass spectrometer equipped with a nano-electrospray ion source (NSI source) was used for the analysis (Thermo Fisher Scientific). An equivalent of 500 ng peptides were resuspended in 0.1 % FA in water and loaded to a C18 pre-column (1 cm × 150 μm, 1.9um particle size, 100 Å pole size, Dikma Technologies, USA) and analyzed by a self-packed C18 analytical column (1.9um particle size, 100 Å pole size, 15 cm × 150 μm). A 60 min LC gradient [solvent A: 0.1 % formic acid(Fluka, Saint Louis, USA) in water, solvent B: 80 % ACN (ThermoFisher Scientific, Waltham, USA), 0.1 % formic acid in water] to 4 % B at 0 min, 7 % B at 1 min, 13 % B at 6 min, 25 % B at 36 min, 45 % B at 52 min, 95 % B at 53 min held until 60 min at a flow rate of 600 nl/min was used to separate peptides for all LC-MS/MS analysis.

**Fig. 1:**
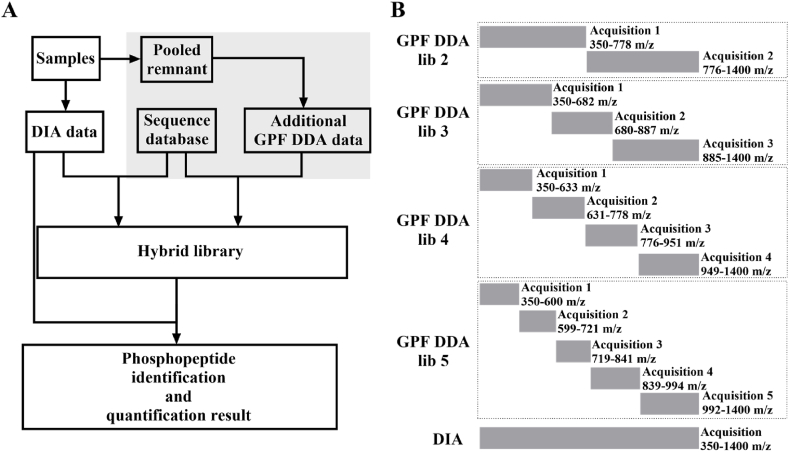
Schematic diagram of GPF DDA hybrid DIA analysis of phosphoprteome and design of GPF DDA data acquisitions. A. Diagram illustrating GPF DDA hybrid DIA analysis of phosphoprteome. Enriched phosphopeptide samples were analyzed using DIA. The remaining samples were combined to create a mixture, which underwent additional GPF DDA analysis (highlighted in light grey). The DIA runs were subjected to library-free database search to generate a predicted library. The GPF DDA runs were used to construct a DDA spectrum library. A hybrid library was built and employed to reanalyze the DIA data and finalizing the identification of phosphopeptides. B. MS1 scan ranges for GPF DDA and DIA data acquisition. The full MS1 range of 350–1400 *m*/*z* was covered by a single DIA acquisition for DIA runs or by 2–5 GPF DDA acquisitions for hybrid library construction as depicted.

Eluted peptides were sprayed by the emitter coupled to the analytical column kept at 60 °C and introduced to Q Exactive HF mass spectrometer under high voltage of 2.2 kV. For DIA analysis, ms1 was scanned at a *m*/*z* rang of 350–1400 with a resolution of 120,000 at m/z 200 and AGC target was set to 3E6. The maximum of ion injection time was 80 ms. Twenty-seven ms2 scans with variable window widths designed by the window scheme wizard embedded in software EncyclopeDIA [20] were followed with HCD energy of NEC 27 %. The overlap of neighboring scans was 1 th. The resolution of ms2 was 30000 at m/z 200 and ACG target was 1E6. For GPF DDA analysis, ms1 scan ranges varied (see Fig. 1B of results for details). The top 20 abundant ions were selected and fragmented with HCD of NEC 27 %. The orbtirap resolution was 120,000 and 15,000 at m/z 200 for ms1 and ms2, and AGC target was 3E6 and 5E4. The maximum ion injection time was 80 ms and 20 ms, respectively. Dynamic exclusion was on and set to 15 s.

### 2.4 Raw data processing and bioinformatics

The mass spectrometry raw data were searched against Uniprot human (version 2022/8) or mouse (version 2022/10) proteome database using software Spectronaut (version 16.1) (Biognosys, Switzerland). GPF-DDA database search and library generation were conducted by Pulsar search engine through Spectronaut. The digestion enzyme was Trypsin/P with a maximum of two missed cleavages. Cysteine carbamidomethylation was set as a fixed modification, phosphorylation on serine/threonine/tyrosine, methionine oxidation and acetylation of the protein N-terminus were set as variable modifications, with a maximum of five modification sites per peptide. The best N fragments per peptide was 3–25. PTM localization filter was on and set to 0.75. Library based DIA search was performed using Spectronaut against the GPF DDA libraries with PTM probability cutoff checked and set to 0.75. Direct DIA search was also performed using Spectronaut. The digestion enzyme was Trypsin/P with a maximum of two missed cleavages. Cysteine carbamidomethylation was set as a fixed modification, phosphorylation on serine/threonine/tyrosine, methionine oxidation and acetylation of the protein N terminus were set as variable modifications with a maximum of five modification sites per peptide. PTM localization filter and PTM localization cutoff were both set to 0.75. The GPF-DDA hybrid DIA search was conducted in a manner similar to direct DIA described above using spechtronaut, and including GPF DDA data as additional library runs to build a hybrid library for the DIA search. All data were filtered at 1 % false discovery rate (FDR) at peptide spectrum match (PSM)/Precursor and Peptide.

The Spectronaut precursor-level quantification data was transformed into a phosphosite level quantification using Perseus with plugin peptide collapse (https://github.com/AlexHgO/Perseus_Plugin_Peptide_Collapse) [6]. R statistical language was used to process data with R/Biocmanger packages including tidyverse, corrplot, DMwR2, ggseqlogo, seqinr and limma [[21], [22], [23], [24]]. Phosphosites having at least two quantitative values from three biological replicates were analyzed for differential expression. The expression data underwent log2 transformation and normalized to achieve a uniform median. The fold change of phosphosite expression was calculated by dividing the mean expression values of replicates in one condition by those in another. Differential expression analysis was conducted using R software package limma [22]. Differentially expressed phosphosites were defined as having an adjusted p-value of less than 0.05 and a fold change greater than 2 in comparisons among any two of three ALI conditions. DAVID Bioinformatics Resources tool was used to retrieve GO and KEGG pathway enrichment of phosphoproteins [25,26]. The online software MoMo version 5.5.0 (https://meme-suite.org/meme/tools/momo) was utilized to discover sequence motifs [27].

### 2.5 Quality control of LC-MS/MS

A standard HEK293 peptide samples was subject to LC-MS/MS analysis with DDA mode every 48 h. A similar setting to GPF-DDA was used for QC runs with some modification. The LC gradient was to 4 % B at 0 min, 7 % B at 1 min, 13 % B at 7 min, 25 % B at 45 min, 45 % B at 67 min, 95 % B at 68 min held until 75 min at a flow rate of 600 nl/min and the MS1 scan range was 300–1400. The top 25 abundant ions were selected for ms2 scans. Raw QC data was analysis with MaxQuant (version 2.2.0.0) against Uniprot human (version 2022/8) proteome database. The digestion enzyme was trypsin/P, with a maximum of two missed cleavages. Cysteine carbamidomethylation was set as a fixed modification, and methionine oxidation and acetylation of the protein N-terminus were set as variable modifications, with a maximum of five modification sites per peptide. False discovery rate was controlled to 1 % for PSMs, peptides and proteins. LFQ quantification and match between run were enabled. The other parameters were set to default values. The LFQ data for proteins was used for Pearson correlation analysis.

## 3 Results

### 3.1 Workflow for GPF-DDA hybrid DIA phosphoproteome and MS1 scan coverage of GPF-DDA acquisitions

To fully utilize the leftover phosphopeptides in DIA analysis of phosphoproteome, as shown in Fig. 1A, the remaining phosphopeptides from each sample were pooled together to create a mixture. This mixture was analyzed to create an extra DDA spectrum library for the DIA data by injecting it multiple times, with each injection scanning a different ms range (GPF-DDA). By merging this DDA library with the predicted library from direct DIA database search of DIA data, a hybrid library was constructed. The identification of phosphopeptides from the DIA data was then accomplished by searching against the hybrid library. The number of GPF DDA runs can range from two to multiple, depending on the quantity of pooled phosphopeptides. A design of ms1 coverage of two to five GPF-DDA acquisitions was demonstrated in Fig. 1B. The scanning window width for each GPF DDA acquisition was optimized based on the ion distribution of phosphopeptides across a full MS1 scan range of 350–1400 *m*/*z*.

### 3.2 Comparison of direct DIA and GPF-DDA hybrid DIA in phosphoproteome analysis

**Fig. 2:**
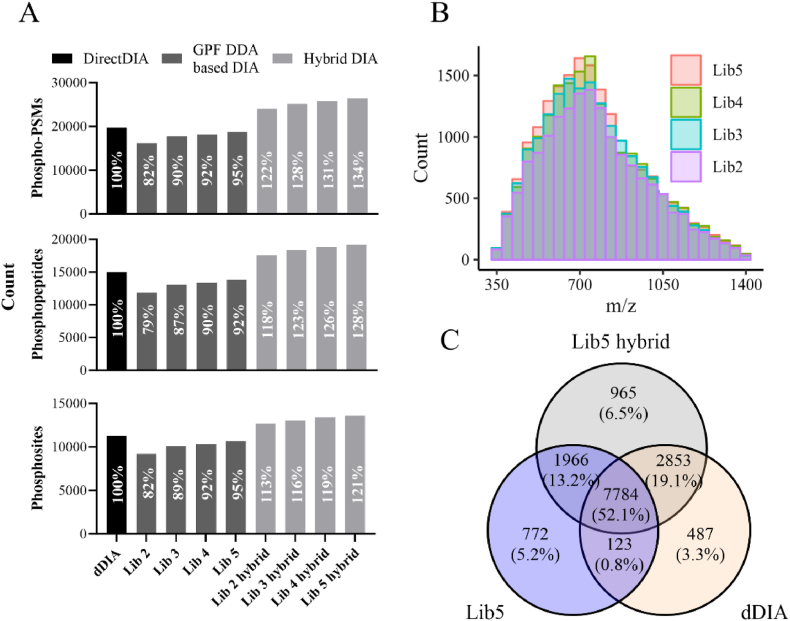
Identification of phospho-PSMs, -peptides, and -sites in different DIA methods: direct DIA (dDIA), 2-5GPF DDA based DIA (Lib 2–5), and hybrid DIA (Lib 2–5 hybrid). A. Bar graph showing the number of phospho-PSMs (upper panel), phosphopeptides (middle panel), and phosphosites (lower panel) identified in the libraries and DIA data with the relevant libraries. Two identifications out of three DIA replicates were included in the phosphosite identification. The percentages of identification for various search strategies compared to direct DIA identification were shown on the graph. B. Histogram illustrating the phospho-PSMs identified in the GPF DDA libraries. C. Venn diagram comparing the phosphosites identified in Lib5 hybrid DIA, Lib5 based DIA and direct DIA.

The GPF-DDA data can be used solely to create a spectrum library or generate a hybrid library that includes both DDA spectrum and predicted spectum extracted from DIA data. Along with the increase in number of GPF-DDA acquisitions, more phospho-precursors are identified in GPF-DDA libraries, expanding the hybrid libraries to cover a wider range of phospho-precursors (Fig. 2A, upper panel). Additionally, the identification of phosphopeptides from the DIA data is improved (Fig. 2A, middle panel), with the maximum identification of 25,000 phospho-precursors, 20,000 phosphopeptides and 13,000 phosphosites (Fig. 2A lower panel). This increased identification could be attributed to the identification of more phospho-precursors throughout the *m*/*z* range of scanning (Fig. 2B–Supplementary Table S1). Compared to dDIA, GPF-DDA based DIA preferentially covers more phospho-precursors in the low *m*/*z* range (Supplementary Fig. S1A). Around 2000 and 2800 phosposites were exclusively identified in GPF-DDA-based DIA search and direct DIA search, respectively (Fig. 2C). The hybrid search combines GPF-DDA and direct DIA searches to achieve the maximum identification, identifying 2931 additional phosphosites associated with 2874 phosphopeptides compared to direct DIA. Of these phosphopeptides, 45 % were isoforms of those identified in direct DIA, while 55 % were newly discovered (Supplementary Fig. S1B). Regardless of the search strategy employed in DIA data analysis, a significant overlap in the identification of phosphosites between replicates was observed. (Supplementary Fig. S2).

**Fig. 3:**
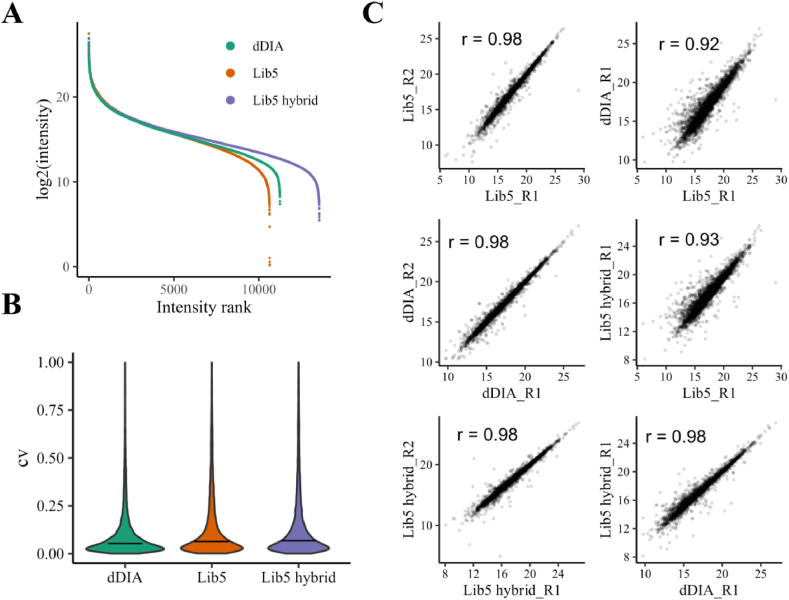
Phosphosite quantification of direct DIA, GPF DDA-based DIA and hybrid DIA. A. Scatter plot depicting phosphosite intensities against their intensity rank in direct DIA, Lib5 GPF-DDA-based DIA and Lib5 hybrid DIA. B. Violin plot illustrating coefficient of variance for phosphosites in direct DIA, Lib5 GPF DDA-based DIA and Lib5 hybrid DIA. A horizontal line indicating the median CV was shown in the plot. The median CV was 0.053 (dDIA), 0.064 (Lib5) and 0.067 (Lib5 hybrid). C. Scatter plot comparing phosphosites between replicates and different DIA search strategies. A Pearson correlation was shown on the graphs.

Compared to the direct DIA search, GPF DDA-based DIA search exhibited a wider dynamic range. This improvement also benefitted the hybrid DIA search, allowing it to detect more low-abundance phosphosites (Fig. 3A). Supplementary Fig. S3 presents example chromatograms of three low-abundant phospho-precursors captured in hybrid DIA. In terms of coefficient of variance (CV) of phosphosites, direct DIA, GPF DDA-based DIA and hybrid DIA search showed a similar distribution of CVs (Fig. 3B). A high Pearson correlation (r > 0.9) was observed between replicates within direct DIA, GPF DDA-based DIA and GPF-DDA hybrid DIA database search (Fig. 3C, Supplementary Fig. S4).

### 3.3 GPF-DDA hybrid DIA phosphoproteome analysis of direct and indirect ALI

**Fig. 4:**
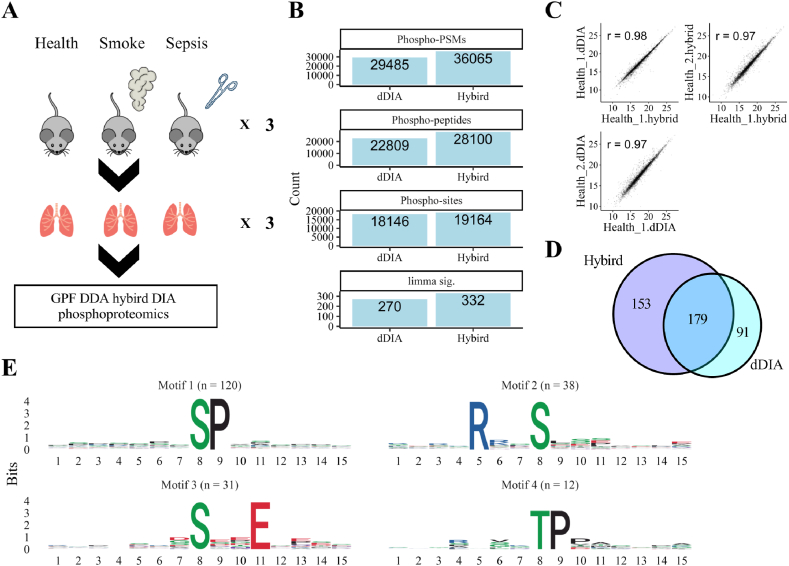
Phosphoproteome analysis of mouse ALI. A. Schematic workflow for the phosphoproteome analysis. Lung tissue from smoke- and sepsis-induced ARDS/ALI, as well as healthy lung tissue (each n = 3) were collected and subjected to GPF-DDA hybrid DIA phosphoproteome analysis. B. Bar plot for identification of phospho-PSMs, phospho-peptides, phosphosites and differentially expressed phosphosites. C. Scatter plot for phosphosites of samples from healthy tissue identified in dDIA and hybrid DIA. Pearson correlation was shown on the graphs. D. Venn diagram for differentially expressed phosphosites in dDIA and hybrid DIA. E. Sequence logo for motifs identified from the differentially expressed phosphosites. The 8th position in logos represents the phosphosites. The number of phosphosites matching the motif is displayed on top of each motif logo.

GPF-DDA hybrid DIA analysis comprising three GPF-DDA was employed in the analysis of phosphoproteome of mouse lungs upon ALI induced by pulmonary direct (smoke) and indirect (sepsis) risks, as well as health lungs (Fig. 4A). More than 28,000 phosphopeptides and 19,000 phosphosites were identified in the hybrid DIA search which outperforms direct DIA search strategy (Fig. 4B). Quantified phosphosites were well correlated between different DIA search strategies and among replicates (Fig. 4C, Supplementary Fig. S5A). Similar distribution of CVs was also observed among two DIA search strategies (Supplementary Fig. S5B). A total of 332 phosphosites were differentially expressed in the hybrid DIA search by employing limma significance test (Fig. 4B). A considerable overlap of differentially expressed phosphosites (DEPsites) was shown when comparing with direct DIA and more DEPsites were identified in the hybrid DIA search (Fig. 4D). Conserved motifs may exist around the phosphosites. Four sequence motifs were discovered among the sequences near the 332 DEPsites, with Proline (P), Serine (S) and Glutamic Acid (E) being the most prevalent residues around serine and threonine phosphorylation sites (Fig. 4E).

**Fig. 5:**
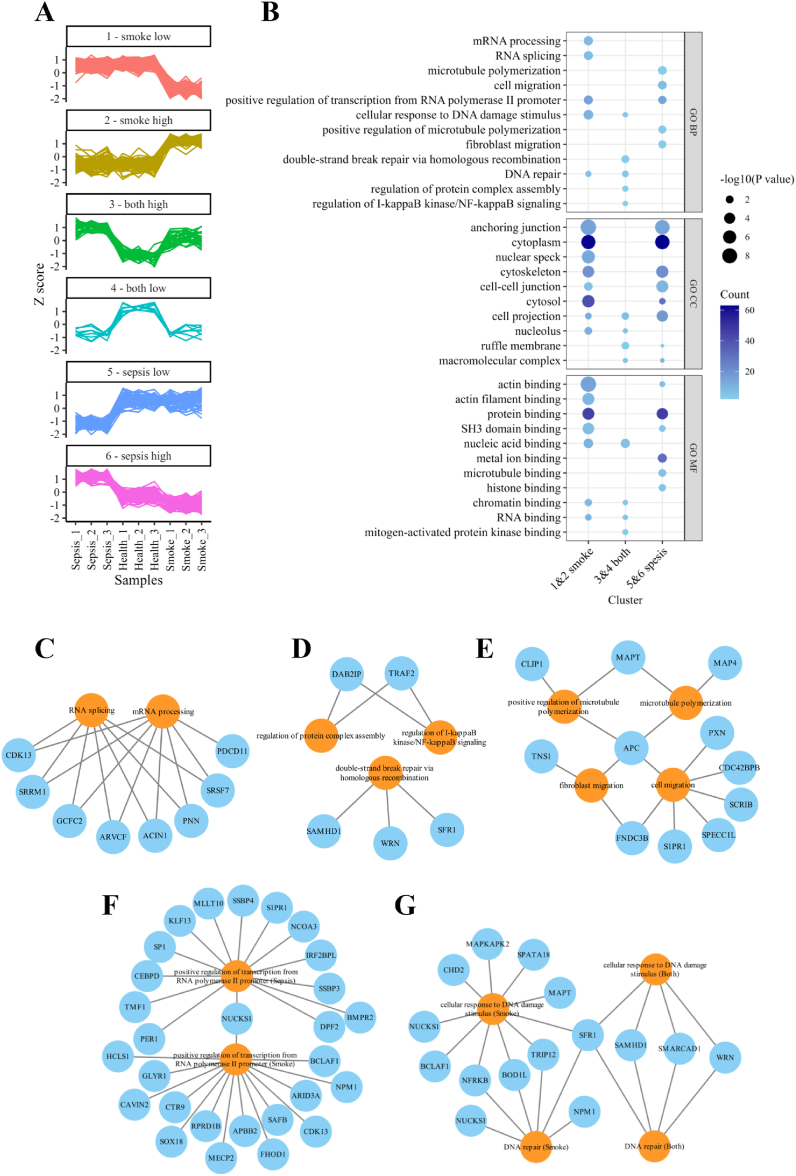
Clustering and GO annotation of proteins of differentially expressed phosphosites. A. Expression profile of 6 clusters of phosphosites displaying similar expression trends. Each line represents the expression profile of a phosphosite across samples. Phosphosites in each cluster exhibit a similar trend of high (or low) expression in either sepsis or smoke-induced ALI or both, as indicated in the title of each cluster. B. GO annotation of proteins of DEPsites in clusters of A. Phosphosites in cluster 1 and 2 shown low and high expression in smoke-induced ALI; Phosphosites in cluster 3 and 4 shown high and low expression in both smoke- and sepsis-induced ALI; Phosphosites in cluster 5 and 6 shown low and high expression in sepsis induced ALI. BP, biological process; CC, cellular component; MF, molecular function. C. D. E. Networks showing uniquely enriched GOBP terms in ALI induced by smoke (C), both smoke and sepsis (D), and sepsis alone (E), along with their associated proteins with DEPsites. F. G. Networks showing shared GOBP terms in ALI induced by smoke and sepsis separately (F), smoke and both smoke and sepsis (G), along with their associated proteins with DEPsites. Nodes in sky blue indicate proteins of DEPsites and color orange indicates GO BP terms. Edges connect terms and their involved proteins.

Six clusters of phosphosites with similar expression trends were identified among the differentially expressed phosphosites using hierarchical clustering (Fig. 5A). Cluster 1 and 2 display low and high expression in smoke-induced ALI, while cluster 5 and 6 exhibit low and high expression in sepsis-induced ALI. Phosphosites in cluster 3 and 4 show low and high expression in both smoke- and sepsis-induced ALI when compared to the health control. The Gene Ontology (GO) analysis was performed on proteins of the 6 clusters to retrieve their enriched items. GO biological process (BP) unique to smoke, sepsis induced ALI or both was identified (Fig. 5B upper panel). mRNA processing, RNA splicing (Fig. 5C) was uniquely enriched in proteins of DEPsites in smoke-induced ALI. Proteins of DEPsites in both smoke- and sepsis-induced ALI were distinctively enriched in double-strand break repair via homologous recombination, regulation of protein complex assembly and regulation of I-kappaB kinase/NF-kappaB signaling (Fig. 5D). While proteins of DEPsites in sepsis-induced ARDS/ALI were enriched in microtubule polymerization, cell migration, positive regulation of microtubule polymerization and fibroblast migration (Fig. 5E). Positive regulation of transcription from RNA polymerase II promoter was enriched in smoke and sepsis induced ARDS/ALI separately. However, the involved proteins were quite different (Fig. 5F). This also happened to cellular response to DNA damage stimulus and DNA repair, two GO BP terms enriched in smoke induced ALI and both smoke and sepsis induced ALI (Fig. 5G). In GO cellular component (CC) analysis, proteins of DEPsites in smoke and sepsis induced ALI (cluster 1, 2, 5 and 6) were enriched in anchoring junction, cytoplasm, cytoskeleton, cell-cell junction, cytosol and cell projection, only nuclear speck was uniquely enriched in smoke induced ALI (Fig. 5B middle panel). Proteins of DEPsites in both smoke and sepsis induced ALI (cluster 3 and 4) were enriched in GO CC cell projection, nucleolus, ruffle membrane and macromolecular complex. These proteins of DEPsites mainly possessed binding compacity to different molecules (Fig. 5 B lower panel), such as actin, actin filament, protein, nucleic acid, RNA, metal ion, microtubule and histone.

A standard HEK293 peptide sample was analyzed every 48 h to evaluate instrument performance. The QC runs produced similar numbers of MS, MS/MS, precursors, peptides and proteins, with a median protein CV of 8.7 % (Supplementary Table S2). A minimum Pearson correlation of 0.99 was observed among QC runs (Supplementary Fig. S6).

## 4 Discussion

PTMs play significant roles in regulating activity, localization and interaction of proteins with other cellular molecules such as DNA, RNA and proteins. Systematic study of proteome of PTMs helps to elucidate their bio-functions. The development of algorithm of library-free DIA phosphoproteomics allows identification of phosphoproteome without generation of effort- and sample-consuming DDA libraries become a reality. However, compared to a project-specific DDA spectral library, low percent identification of shared phosphopeptides in direct DIA was reported in some cases, suggesting further improvement is needed in direct DIA [28]. Hybrid library constructed from DDA and DIA data has been demonstrated effective in promoting depth of DIA analysis of proteome [29]. Gas phase fractionation shown its advantages in DIA and DDA analysis with spend of only extra LC-MS/MS injections [8,9,11]. We optimized the use of gas phase fractionation in hybrid library generation for phosphoproteome. By utilizing a hybrid library containing a GPF DDA library and a predicted DIA library, significant improvement in phosphopeptide identification was achieved. The number of GPF DDA injections can be adjusted based on the amount of leftover phosphopeptides, with a minimum of two GPF DDA injections proven to be adequate for conducting GPF DDA hybrid DIA phosphoproteome analysis. However, a larger number of GPF DDA injections could lead to a deeper phosphoproteome identification. If sufficient leftover phosphopeptides are available, high PH pre-fractionation may be considered for the hybrid library construction, and/or a longer LC gradient can be used to enhance peptide separation to improve phosphoproteome identification. Pooling samples for GPF-DDA runs should only be conducted after the quality of sample be confirmed through a preliminary library-free analysis of the DIA runs, as pooling may introduce potential contamination.

Next, we proceeded to assess its applicability for samples of ALI. Approximately 1.8 μg of pooled phosphopeptides was remained after DIA analysis, leading us to include three GPF-DDA runs in the analysis. Over 28000 phosphopeptides and 19000 phosphosites were identified using GPF DDA hybrid DIA strategy, which achieved an increase of 23 % and 6 % at phosphopeptide and phosphsite level, respectively, compared to the standard direct DIA. Direct and indirect ARDS/ALI are different in proteome and metabolome of patients’ plasma and urine, manifestations of lung alterations and response to treatments [[30], [31], [32]]. However, no report has yet compared the phosphoproteome of ARDS/ALI caused by the two distinct type of factors and the health control group. In this study, phosphosites up- or down-regulated in lungs specific to smoke or sepsis induced ARDS/ALI, or both, were identified, suggesting phosphorylation has distinct roles in ARDS/ALI of two different subphenotypes. Phosphorylation of proteins involving RNA splicing were specifically enriched in smoke direct induced ALI. In SARS-CoV-2 direct induced ARDS/ALI, host mRNA splicing was also globally inhibited [33,34]. Diffuse injury to endothelial cells is prominent in indirect ARDS/ALI, and depolymerization of microtubules contributes to endothelial dysfunction in ALI [13,35]. In our work, phosphorylation of proteins of microtubule polymerization was enriched in indirect sepsis induced ALI. During sepsis-ALI, decrease of RNA polymerase II density in lung were observed and corelated to expression of proteins [36]. Moreover, we observed changes of phosphorylation involving regulation of transcription from RNA polymerase II promoter in both smoke or sepsis induced ALI. Sequence motifs may indicate the specific recognition by enzymes that catalyze phosphorylation in ALI. Four sequence motifs were identified among the DEPsites. Further studies are required to explore the enzymes and kinases that recognize these motifs and their roles in ALI across two different subphenotypes.

Computational algorithm based direct DIA and library based DIA are two main DIA PTMs analysis strategies [37,38]. In this work, we presented GPF DDA hybrid DIA phosphoproteome analysis workflow utilizing the leftover sample to promote depth of direct DIA analysis of phosphoproteome. This approach could also be extended to DIA analysis of other PTMs.

## CRediT authorship contribution statement

**Zhiwei Tu:** Writing – original draft, Methodology, Investigation, Formal analysis, Conceptualization. **Yabin Li:** Methodology, Investigation, Formal analysis, Data curation. **Shuhui Ji:** Methodology, Formal analysis, Data curation. **Shanshan Wang:** Investigation, Formal analysis, Data curation. **Rui Zhou:** Investigation, Data curation. **Gertjan Kramer:** Methodology, Formal analysis. **Yu Cui:** Validation, Supervision, Methodology, Conceptualization. **Fei Xie:** Writing – review & editing, Validation, Resources, Project administration, Conceptualization.

## Ethics statement

This study was reviewed and approved by the Institutional Animal Care and Use Committee at Beijing Institute of Radiation Medicine with the approval number: IACUC-DWZX-2020-534, dated December 12, 2020.

## Data availability statement

The mass spectrometry proteomics data have been deposited to the ProteomeXchange Consortium (https://proteomecentral.proteomexchange.org) via the iProX partner repository with the dataset identifier PXD051290.

## Funding

This study was supported by the 10.13039/501100001809National Natural Science Foundation of China (No. 61976223).

## Declaration of competing interest

The authors declare that they have no known competing financial interests or personal relationships that could have appeared to influence the work reported in this paper.

## Abbreviations

DIA: Data independent acquisition
DDA: data dependent acquisition
PTMs: post translational modifications
GPF: gas phase fractionation
ALI: acute lung injury
CLP: cecal ligation and puncture
