# Supplementary material for: Gas-phase fractionation DDA promotes in-depth DIA phosphoproteome analysis
Source: Heliyon. 2025 Jan 14;11(2):e41928. doi: 10.1016/j.heliyon.2025.e41928 (PMC11787513; doi:10.1016/j.heliyon.2025.e41928)
Supplement: Multimedia component 1 [file mmc1.docx]

Supplementary Materials

**
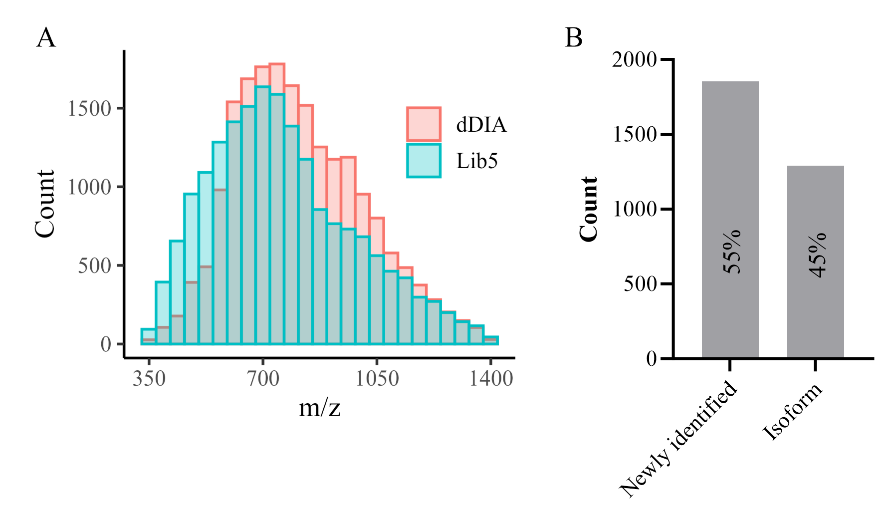
**

**Supplementary Figure S1.** Histogram of phospho-precursors identified in dDIA and lib5 based DIA (A) and bar graph of phosphopeptides identified solely in lib5 hybrid DIA (B). In B, 45% phosphopeptides were isoforms of phosphopeptides identified in direct DIA, while 55% were newly discovered.


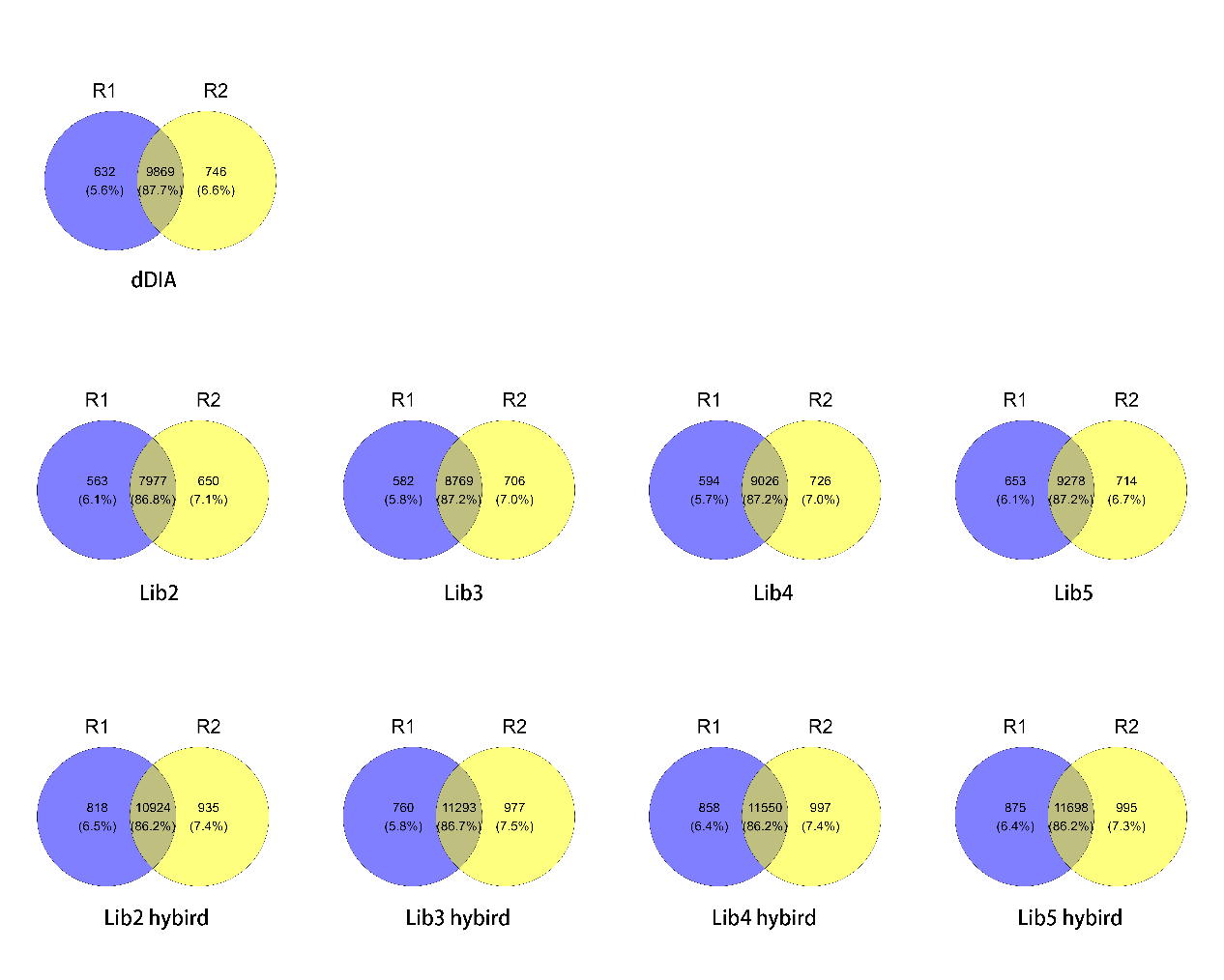


**Supplementary Figure S2.** Venn diagram for overlapped phosphosites between the technical replicates of direct DIA, lib2-lib5 GPF DDA base DIA and lib2-lib5 hybrid DIA.


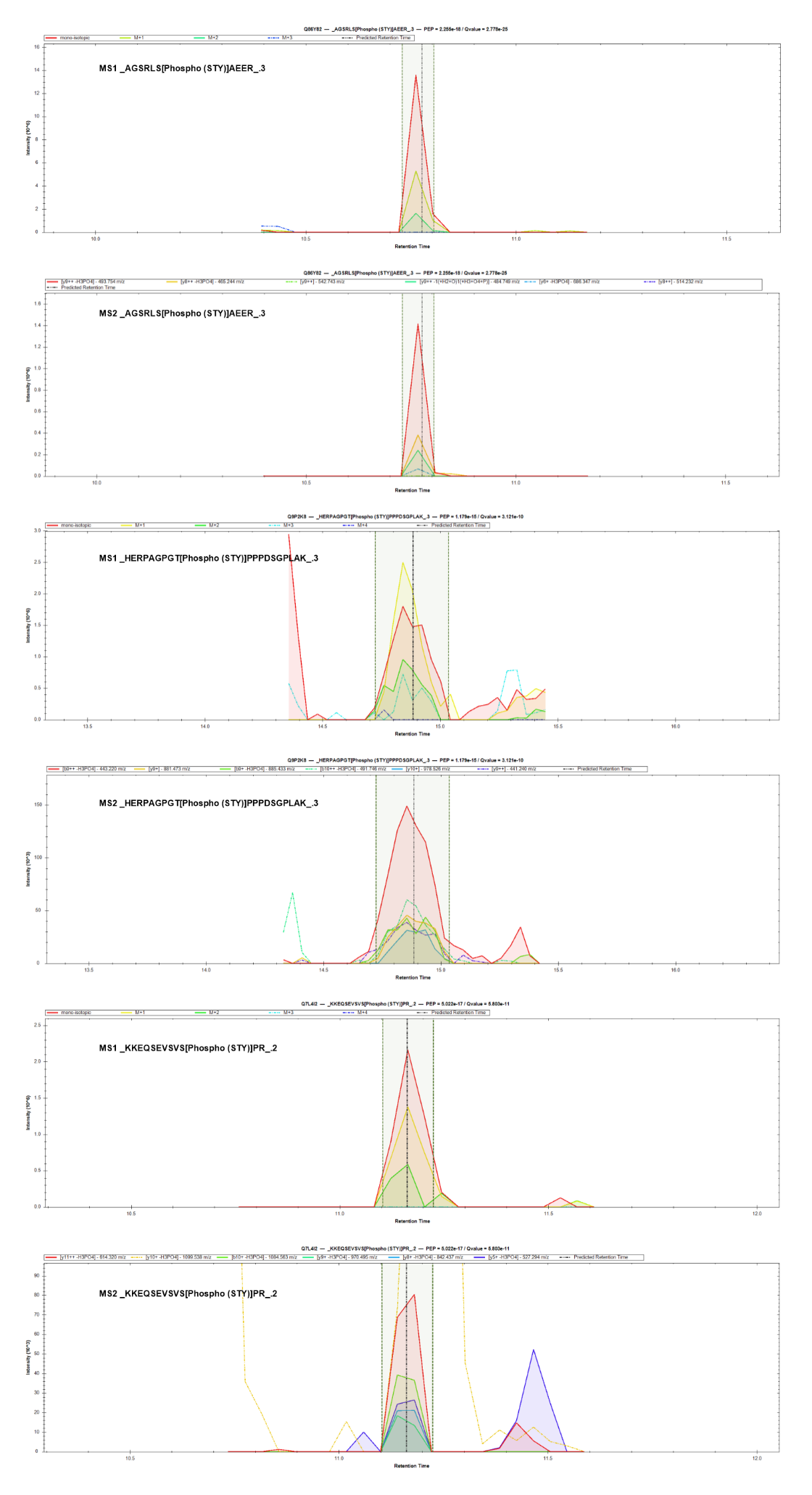


**Supplementary Figure S3.** MS1 and MS2 chromatograms of three low-abundant phospho-precursors detected in hybrid DIA but not in dDIA.


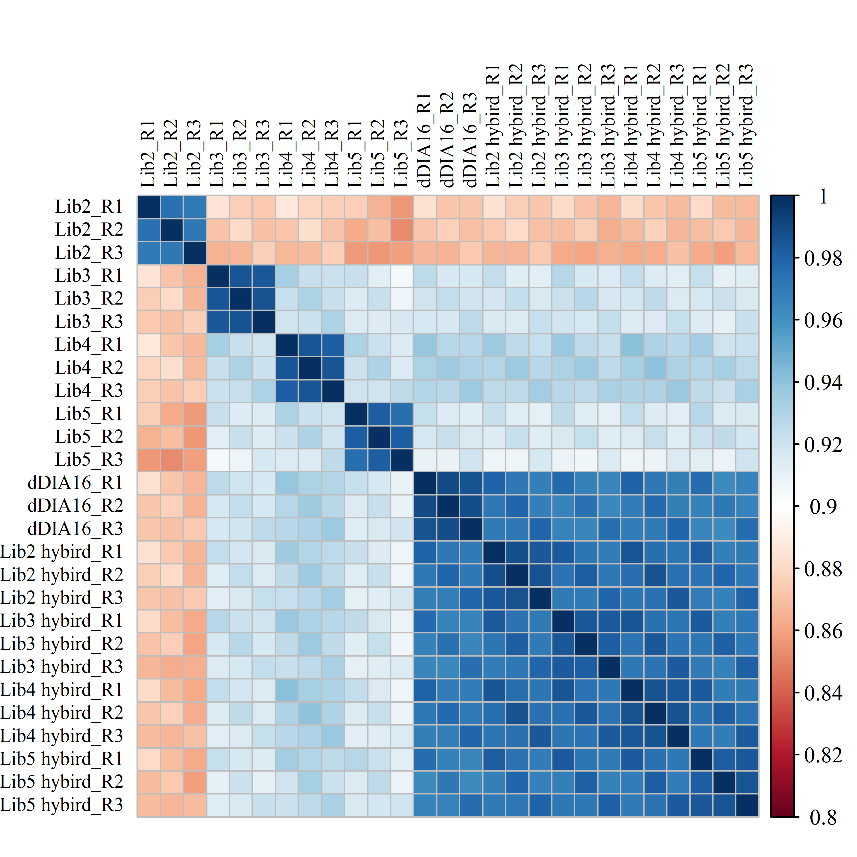


**Supplementary Figure S4.** Pearson correlation for phosphosites identified from direct DIA, GPF-DDA based and hybrid DIA. Three replicates were included for every search strategy.


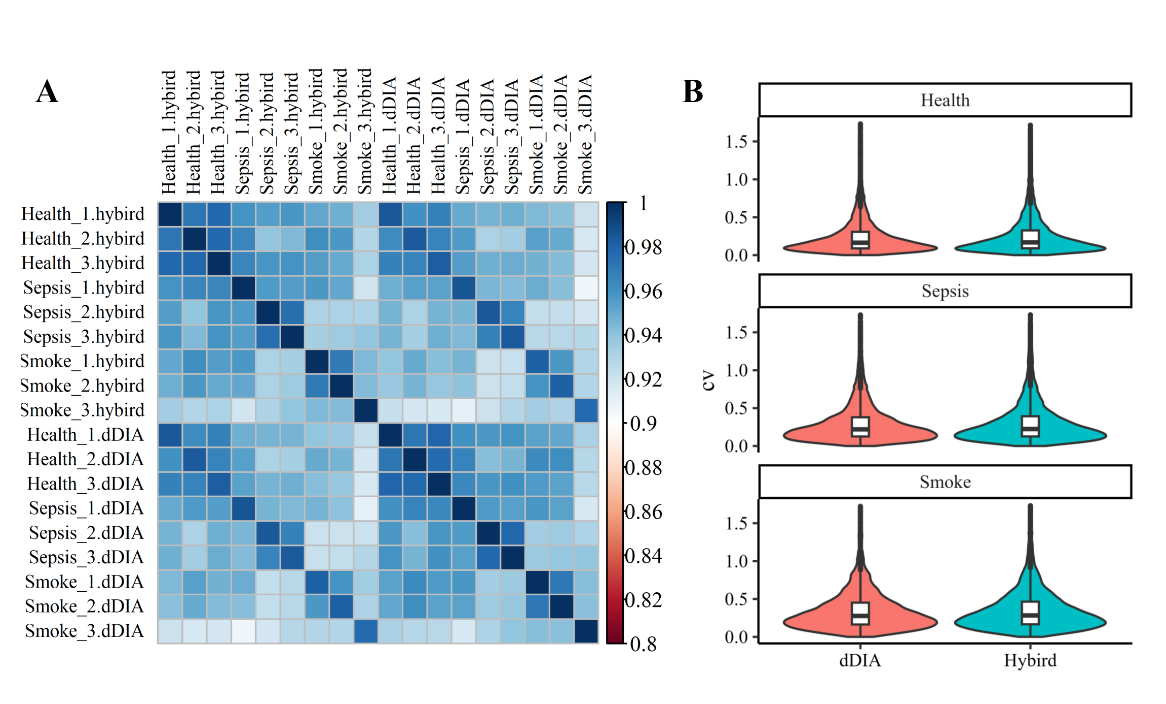


**Supplementary Figure S5.** Person correlation and CVs of phosphosites identified with two DIA strategies from health lungs, as well as smoke- and sepsis-induced ALI lungs. A. Person correlation of phosphosites. B. Violin plot of phosphosites’ CVs. Boxplots were shown within the violins. dDIA, direct DIA; hybrid, GPF-DIA hybrid DIA.


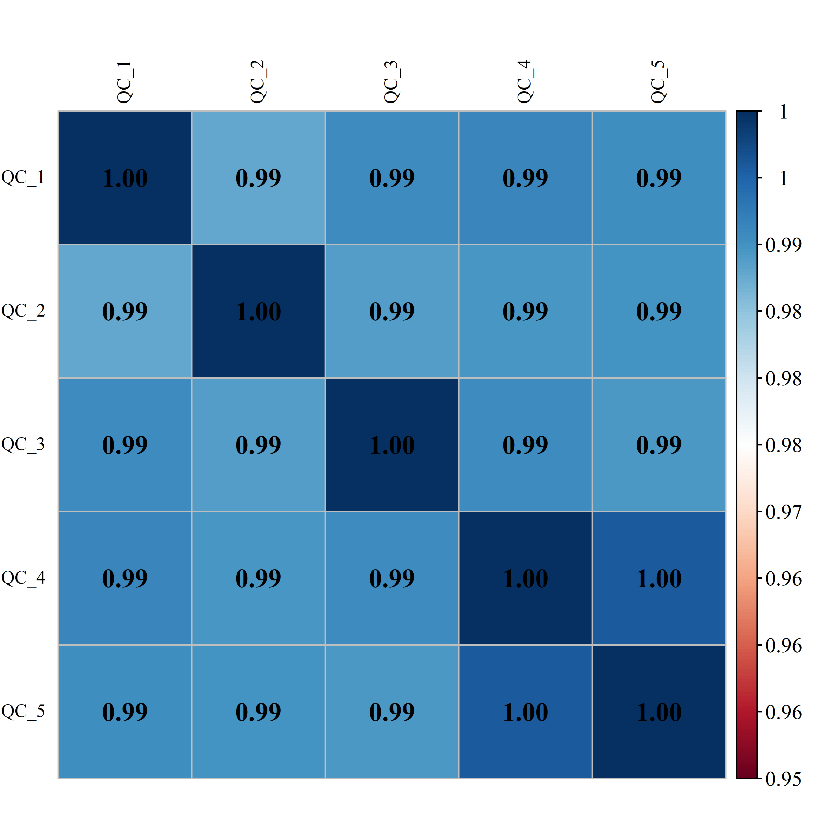


**Supplementary Figure S6.** Person correlation of proteins from QC runs.

**Supplementary Table S1.** Identification summary for dDIA, Lib 2-5 based DIA and Lib 2-5 hybrid DIA.

|  | phospho-PSMs | phosphopeptides | | phosphosites |
| --- | --- | --- | --- | --- |
| dDIA | 19685 | 14944 | 11247 | |
| Lib 2 | 16122 | 11838 | 9190 | |
| Lib 3 | 17776 | 13030 | 10057 | |
| Lib 4 | 18142 | 13386 | 10346 | |
| Lib 5 | 18732 | 13802 | 10645 | |
| Lib 2 hybrid | 24027 | 17561 | 12677 | |
| Lib 3 hybrid | 25182 | 18328 | 13030 | |
| Lib 4 hybrid | 25755 | 18775 | 13405 | |
| Lib 5 hybrid | 26353 | 19176 | 13568 | |

**Supplementary Table S2.** Identification summary of standard HEK293 peptides.

|  | MS | MS/MS | PSMs/Purcursors | Peptides | Proteins | Median CV [%] (proteins) |
| --- | --- | --- | --- | --- | --- | --- |
| QC_1 | 3645 | 71457 | 34048 | 34448 | 4058 | 8.7 |
| QC_2 | 3665 | 72328 | 35941 | 34596 | 4047 |  |
| QC_3 | 3595 | 73479 | 34061 | 33299 | 4023 |  |
| QC_4 | 3532 | 73109 | 32628 | 33822 | 4013 |  |
| QC_5 | 3483 | 73931 | 32604 | 33831 | 4026 |  |
